# Atypical Sjögrenʼs Syndrome Initially Presenting as Lymphocytic Interstitial Pneumonitis followed by Immune Thrombocytopenia

**DOI:** 10.1155/2021/6681590

**Published:** 2021-03-15

**Authors:** Maham Mehmood, Abhishrut Jog, Masooma Niazi, Arlene Tieng, Giovanni Franchin

**Affiliations:** ^1^Department of Internal Medicine, BronxCare Health System, Bronx, NY, USA; ^2^Department of Pathology, BronxCare Health System, Bronx, NY, USA; ^3^Icahn School of Medicine at Mount Sinai, New York, NY, USA; ^4^Hofstra University School of Medicine, Long Island, NY, USA

## Abstract

**Background:**

Sjögrenʼs syndrome is an autoimmune disease characterized primarily by decreased exocrine gland function leading to eye and mouth dryness. Extraglandular manifestations occur less frequently. *Case Report*. A 74-year-old man with hypertension was admitted with productive cough and fever. On physical examination, he had bilateral lower lung decreased breath sounds. A chest radiograph showed bibasilar patchy infiltrate. Laboratory studies revealed hemoglobin of 11.9 g/dL, white blood cell count of 16,000/uL, and platelet count of 250,000/uL. Empiric antibiotic therapy was begun for suspected community acquired pneumonia, and then he was discharged home. However, his cough recurred. Chest computed tomography demonstrated adenopathy throughout the mediastinum and multiple ill-defined patchy groundglass opacities with a lower lobe prominence. He underwent a transbronchial biopsy to rule out malignancy; however, it showed lymphocytic interstitial pneumonitis. Antinuclear antibody was 1 : 80 homogeneous, and anti-SSA antibody was 6.3 AI (normal <1.0 AI). The patient was treated with prednisone 20 mg/day with marked improvement in his symptoms. Repeat chest computed tomography showed decreased groundglass opacities and decreased mediastinal lymph nodes. After more than a year, he was readmitted due to petechiae on his buccal mucosa and a platelet count of 2000/*μ*L. The patient was started on prednisone 80 mg/d and intravenous immunoglobulin 80 g/d for 2 consecutive days. The platelet count eventually increased to 244,000/*μ*L.

**Conclusion:**

We report a rare presentation of Sjogrenʼs syndrome manifesting as acute lymphocytic interstitial pneumonitis and followed by immune thrombocytopenia. Both extraglandular manifestations responded well to corticosteroid therapy.

## 1. Background

Sjögren's syndrome is a chronic systemic autoimmune disorder commonly characterized by dry eyes and dry mouth. It has an incidence rate of 7/100,000 persons annually [1]. In addition to the anti-SSA antibody, the oral and ocular symptoms are assessed in the most recent classification criteria for primary Sjögren's syndrome [2]. However, extraglandular features may occur in a third of the cases and sometimes they are the presenting signs. These can include Raynaud phenomenon or cutaneous vasculitis, arthralgia or myopathy, interstitial lung disease (ILD), primary biliary cholangitis, interstitial nephritis, peripheral neuropathy, or lymphoma [3]. ILD is seen in only 9–20% cases of Sjögren's syndrome, and of the types of ILD, lymphocytic interstitial pneumonitis (LIP) is less frequently identified [4]. LIP was first described in 1966 [5]. As opposed to pulmonary manifestations in Sjögren's syndrome, immune thrombocytopenia (ITP) is not as well-recognized a feature. To the best of our knowledge, we know of no case report of LIP and ITP in Sjögren's syndrome. We present one such case of Sjögren's syndrome that presented as LIP and then developed ITP.

## 2. Case Report

**Figure 1:**
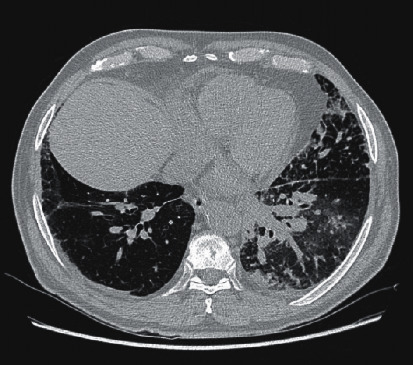
Computed tomography of chest showing transverse view of lungs with multiple patchy ground glass opacities with predominant lower lobe involvement, more than a month after initial presentation.

**Figure 2:**
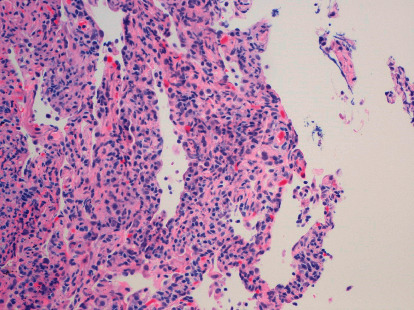
Pathological findings of lymphocytic interstitial pneumonitis. High-power analysis of lung tissue shows interstitial septa thickening, infiltration by small- to medium-sized lymphocytes (hematoxylin-eosin, magnification × 200).

A 74-year-old Ecuadorian man who smoked 3 cigarettes a day for a year but quit 15 years prior presented to the Emergency Department (ED) with four days of a productive cough that did not improve with azithromycin. His medical history included hypertension and cataracts. He worked at a clothing factory for years. The body mass index was 24.9 kg/m^2^. Physical examination showed a blood pressure of 154/86 mmHg, pulse of 115 beats/minute, respiration rate of 18/min, oxygen saturation of 95% on ambient air, and temperature of 101°F. Pulmonary auscultation on hospital admission revealed decreased bilateral breath sounds in the lower lung fields. A chest radiograph showed a large rounded opacity in the superior segment right lower lobe and bibasilar patchy infiltrate. The results of laboratory studies revealed a white blood cell count of 16,000/*μ*l with 82.7% neutrophils and 8% lymphocytes, hemoglobin of 11.9 g/dl, and platelet count of 250,000/*μ*l. Blood and urine cultures yielded no growth, and his acute symptoms of fever and cough subsided with ceftriaxone plus azithromycin. He was discharged home three days after admission. However, his productive cough recurred. More than a month later, computed tomography (CT) scan of the chest revealed multiple, approximately 16.4–28.6 mm, enlarged nodes in the mediastinum and hila, diffuse septal thickening, and multiple ill-defined patchy ground glass opacities with a lower lobe predominance (Figure 1). On transthoracic echocardiography, the systolic pressure of the pulmonary artery was 33 mmHg with normal ejection fraction. The rheumatoid factor was negative, and the antinuclear antibody was positive at 1 : 80 with a homogeneous pattern. The patient was readmitted (2 months after the first episode) due to pleuritic chest pain and dyspnea on exertion. Radiography of the chest revealed bilateral prominent lung interstitial markings particularly in the left perihilar areas, and he was empirically covered for hospital-acquired pneumonia with vancomycin and piperacillin/tazobactam. More than a month later, his C-reactive protein (CRP) was <5 mg/l. He was lost to follow-up for five months but returned complaining of xerophthalmia, and artificial tears provided relief. He was again lost to follow-up for another five months and then noted to have weight loss of 25 pounds over 6 months. Follow-up chest CT findings more than a year after his initial admission included worsening septal thickening and ground glass opacities. Transbronchial lung biopsy demonstrated mild thickening of interstitial septae and increased infiltration by lymphocytes, macrophages, and plasma cells (Figure 2) consistent with LIP. No malignant cells were seen.

Then, he developed bilateral metacarpophalangeal swelling and pain and swelling. To treat LIP, he was started on oral prednisone at a dose of 20 mg/day (0.25 mg/kg). His joint symptoms improved markedly. A laboratory workup was positive for the SSA antibody, and he was diagnosed with Sjögren's syndrome. His CRP was elevated (191 mg/l) and complement levels were low (C3 and C4, 40 mg/dl and 1 mg/dl, respectively). He tested negative for anti-SSB, anti-Jo-1, anti Scl-70, anti-neutrophil cytoplasmic, and anti-double-stranded DNA antibodies. Pulmonary function testing did not show obstructive dysfunction. His postbronchodilator forced expiratory volume in one second (FEV_1_) was 3 liters (126% predicted), total lung capacity (TLC) was 5.48 liters (104% predicted), and diffusion capacity for carbon monoxide (DLCO) was 17.8 ml/min/mmHg (82% predicted). The patient complained of dry mouth, which improved with pilocarpine. He was lost to follow-up for four months, during which time he discontinued prednisone after five months. Then, he developed anterior uveitis which was treated successfully with topical prednisolone acetate. A follow-up chest CT scan more than a year after oral prednisone was first started showed bibasilar and lingular interstitial fibrotic changes, decreased prominence of associated interlobular septal thickening and ground glass opacity, and a decrease in the size of the lymph nodes. Prednisone 20 mg/day was resumed and slowly tapered over three months. A month after prednisone was tapered to 10 mg, he presented to the ED with oral mucosal bleeding. His vital signs were unremarkable. Physical examination was significant for a bloody oral cavity, bruises on the right arm and legs, and scattered petechiae on the chest and lower legs. Laboratory tests were remarkable for a platelet count of 2000/*μ*l. Peripheral smear did not show schistocytes. CT head was negative for acute hemorrhage. Further labs, including liver function, lactate dehydrogenase, and vitamin B12 levels, were normal. Hepatitis C antibody, hepatitis B surface antigen, and human immunodeficiency virus were negative. No obvious drug-related causes of thrombocytopenia were identified. Blood and urine cultures were negative. A diagnosis of ITP secondary to Sjögren's syndrome was made. A bone marrow biopsy was not done.

The patient was started on prednisone at 80 mg/day (1 mg/kg/d) and received a total of 12 units of platelet transfusion. He was given intravenous immunoglobulin (IVIG) at 1 g/kg/d for two days. His platelet count improved to 84,000/*μ*l by the sixth day, and oral bleeding resolved. The patient was discharged home, but he was unable to continue the prednisone because the prescription was accidentally not transmitted to the pharmacy. After five days, he was readmitted with oral mucosal bleeding. The platelet count was found to be 3000/*μ*l. He received IVIG at 1 g/kg/d and IV methylprednisolone 62.5 mg twice a day. By the fifth day, his platelet count was 178,000/*μ*l. He was discharged on prednisone 80 mg/day, and it was uneventfully tapered off over four months. He was also started on hydroxychloroquine for musculoskeletal pain. His latest platelet count around six months after the last admission was 254,000 cells/*μ*l.

## 3. Discussion

We present the case of a 74-year-old man with Sjögren's syndrome who developed LIP, arthritis, uveitis, and ITP in addition to the sicca symptoms. The initial presumptive diagnosis of community acquired pneumonia led to further workup after his initial chest X-ray was suspicious for malignancy. A chest CT scan unexpectedly showed multiple enlarged nodes in the mediastinum and hila as well as ground glass opacities in a man who did not have an extensive smoking history. Given his significant weight loss, a transbronchial lung biopsy was performed to evaluate for malignancy or any other underlying pathology. In this case, LIP was diagnosed and that warranted a thorough rheumatologic investigation. Sjögrenʼs syndrome was diagnosed after malignancy was ruled out. Hypocomplementemia can be associated with systemic disease activity, and indeed this patient had involvement of the lungs, joints, eye, and platelets. LIP is characterized by the proliferation of lymphocytes and plasma cells in lung interstitium with lymphoid follicles and germinal centers. Most patients present with dyspnea, cough, and inspiratory crackles on lung exam. LIP is considered benign but can progress to lymphoma or end-stage lung disease. Prednisone can be administered for at least six months, and rituximab has been found to effective [6].

The autoimmune disease more well known to be associated with thrombocytopenia is systemic lupus erythematosus (SLE), so much so that it is one of the elements in its classification criteria [7]. Thrombocytopenia in Sjögren's syndrome is not as well recognized, and it is exceedingly rare to find severe thrombocytopenia (platelet count < 50,000 cells/*μ*l) in Sjögren's syndrome. In a retrospective study by Ramos-Casals et al. of 380 patients with Sjögren's syndrome, severe thrombocytopenia was found in only 0.4% [8].

In 1950 in St. Louis, the hematology fellow William Harrington injected himself with the plasma from a patient with ITP [9]. This led to a drastic drop in his platelets, which supported a role for autoantibodies in the pathophysiology of the disease. ITP in Sjögren's syndrome can be explained by peripheral destruction of platelets by the circulating antibodies. Sjögren's syndrome and SLE, both with circulating antibodies, are more prone to cause such destruction.

Glucocorticoids are the cornerstone of the management of ITP. Of note, this patient developed ITP after the prednisone for his LIP was tapered. Our patient showed a remarkable response with steroids and IVIG. In refractory cases of Sjögren's syndrome with ITP, rituximab or splenectomy can be considered. Our patient suffered from two major extraglandular manifestations that occurred serially, not simultaneously. The administration of a steroid-sparing agent such as rituximab can be considered a potential treatment for his pulmonary and hematologic involvement.

## 4. Conclusion

The classic features of Sjögren's syndrome include xerophthalmia and xerostomia, but they may not always lead to the diagnosis as they may present later in the course of the illness. The initial absence of these features should not deter testing for Sjögren's syndrome, especially if there is suspicious evidence for ILD. Furthermore, Sjögren's syndrome is a rare cause of ITP. LIP and ITP can respond well to steroids. In Sjögrenʼs syndrome, rituximab can be considered with one serious extraglandular organ involvement, in hopes of preventing additional systems to be severely affected.
